# An Exploratory Study on *CLU, CR1* and *PICALM* and Parkinson Disease

**DOI:** 10.1371/journal.pone.0024211

**Published:** 2011-08-30

**Authors:** Jianjun Gao, Xuemei Huang, YikYung Park, Albert Hollenbeck, Honglei Chen

**Affiliations:** 1 Epidemiology Branch of the National Institute of Environmental Health Sciences, Research Triangle Park, North Carolina, United States of America; 2 Department of Neurology, Pennsylvania State University-Milton S. Hershey Medical Center, Hershey, Pennsylvania, United States of America; 3 Nutritional Epidemiology Branch, National Cancer Institute, Rockville, Maryland, United States of America; 4 AARP, Washington, D.C., United States of America; Brigham & Women's Hospital and Harvard Medical School, United States of America

## Abstract

**Background:**

Recent GWAS and subsequent confirmation studies reported several single-nucleotide polymorphisms (SNPs) at the *CLU, CR1* and *PICALM* loci in association with late-onset Alzheimer's disease (AD). Parkinson disease (PD) shares several clinical and pathologic characteristics with AD; we therefore explored whether these SNPs were also associated with PD risk.

**Methodology/Principal Findings:**

791 non-Hispanic Whites cases and 1,580 matched controls were included in the study. Odds ratios (OR) and 95% confidence intervals (CI) were obtained from logistic regression models. rs11136000 at the *CLU* locus was associated with PD risk under the recessive model (comparing TT versus CC+CT: OR = 0.71, 95% CI: 0.55-0.92, p = 0.008) after adjusting for year of birth, gender, smoking, and caffeine intake. Further adjustment for family history of PD and ApoE ε4 status did not change the result. In addition, we did not find evidence for effect modification by ApoE or known PD risk factors. The association, however, appeared to be stronger for PD with dementia (OR = 0.49, 95% CI: 0.27-0.91) than for PD without dementia (OR = 0.81, 95% CI: 0.61-1.06). The two other SNPs, rs6656401 from *CR1*, and rs3851179 from *PICALM* region were not associated with PD (p>0.05).

**Conclusion:**

Our exploratory analysis suggests an association of *CLU* with PD. This exploratory finding and the role of dementia in explaining this finding needs further investigation.

## Introduction

Alzheimer's disease (AD) and Parkinson disease (PD) are the two most common neurodegenerative diseases among the elderly. These two conditions share several clinical and pathological characteristics. For example, they both are clinically progressive and involve abnormal protein depositions [1]. Further, more than half of PD patients eventually develop dementia [2], and conversely, parkinsonian signs are common among Alzheimer patients [3]. Finally, although not entirely consistent [4], preliminary data suggest that individuals with a family history of PD may have a higher risk for AD [5], [6], [7], [8]. These links suggest that AD and PD may have some shared risk factors and/or common pathogenic mechanisms. However, to date, little effort has been made to search for potential genetic or environmental risk factors that may contribute to both diseases.

Recent large genome-wide association studies (GWAS) have reported several novel susceptibility genetic loci for late onset AD, including clusterin (*CLU*) [9], [10], complement component (3b/4b) receptor 1 (*CR1*) [10] and the phosphatidylinositol binding clathrin assembly protein (*PICALM*) [9]. These findings were subsequently confirmed in a large pooled sample of AD cases and controls [11]. We therefore explored the relationship of PD with selected SNPs from the AD GWAS studies (rs11136000 for *CLU*, rs6656401 for *CR1*, and for rs3851179 for *PICALM*) in a large PD case-control study.

## Results

Table 1 shows population characteristics according to case-control status. As expected, Parkinson patients were less likely to be smokers and drank less caffeinated drinks than controls (p<0.0001). Compared with controls, they were also less likely to be *ApoE* ε4 carriers, but more likely to report a family history of PD. Among Parkinson cases, the average age at onset was 65.7±7.4 years and the average age at diagnosis was 66.6±7.4 years. Approximately 19% of Parkinson patients had dementia as reported by their treating physician or by medical record review, and the prevalence of dementia was higher among Parkinson patients diagnosed before 2000 (23.6%) than those diagnosed after 2000 (13.9%).

**Table 1:** Population characteristics of Parkinson Cases and controls.

	Case (%)	Control (%)	P#
All	791	1580	
Men	604 (76.4)	1244 (78.7)	
Women	187 (23.6)	336 (21.3)	0.19
Year of Birth			
1925-1929	283 (35.8)	616 (39.0)	
1930-1934	264 (33.4)	558 (35.3)	
1935-1939	160 (20.2)	264 (16.7)	
1940-	84 (10.6)	142 (9.0)	0.07
Smoking status			<.0001
Never	368 (46.5)	555 (35.1)	
Former	388 (49.1)	916 (58.0)	
Current	24 (3.0)	93 (5.9)	
Missing	11 (1.4)	16 (1.0)	
Caffeine intake (mg/day)	296.5±338.0	359.8±359.9	<.0001
Family History of PD			<.0001
Yes	112 (14.2)	86 (5.4)	
No	672 (85.0)	1356 (85.2)	
Missing	7 (0.9)	138 (8.7)	
Age at diagnosis (n = 781*)	66.6±7.4	NA	
Age at first symptoms (n = 642*)	65.7±7.4	NA	
Year of diagnosis			
Before 2000	363	NA	
2000 and after	428	NA	
CLU: rs11136000			
CC	281 (35.8)	569 (36.1)	
CT	402 (51.2)	746 (47.3)	
TT	102 (13.0)	261 (16.6)	0.05
PICALM: rs3851179			
GG	298 (38.5)	631 (40.4)	
GA	359 (46.3)	716 (45.8)	
AA	118 (15.2)	215 (13.8)	0.52
CR1: rs6656401			
GG	522 (66.8)	1028 (65.5)	
GA	233 (29.8)	493 (31.4)	
AA	27 (3.5)	48 (3.1)	0.66
ApoE: ε4			
ε4 carrier	631 (80.0)	1166 (75.6)	0.02
Non-ε4 carrier	158 (20.0)	376 (24.4)	

# Chi-Square tests for categorical variables, t-tests for continuous variables *number of participants that provided data.

**Figure 1:**
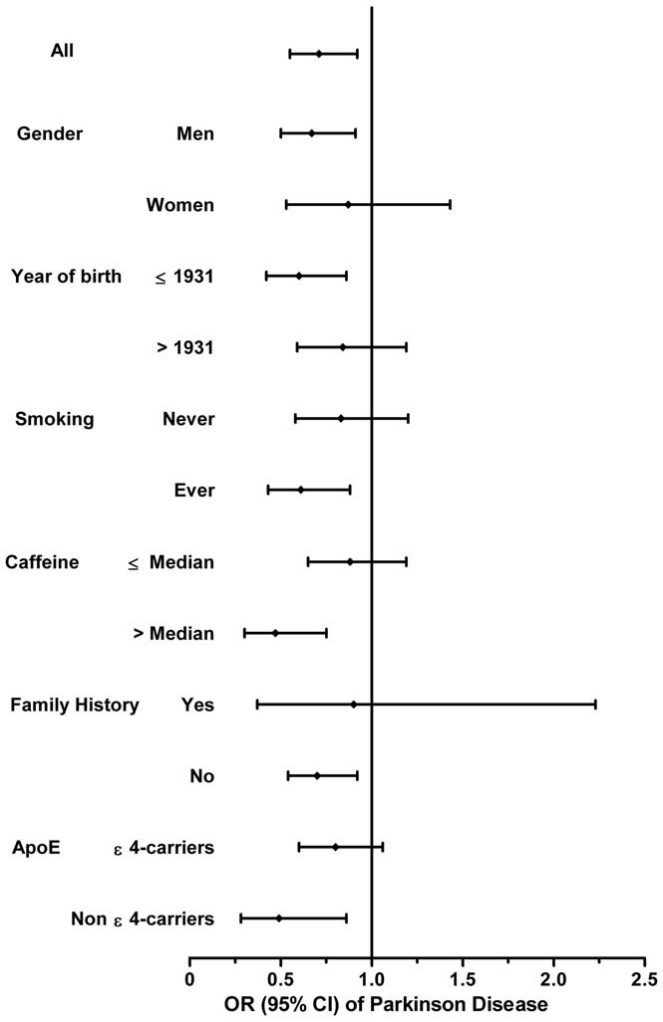
Stratified analysis of the relationship between rs11136000 and PD based on the recessive model (TT vs TC/CC), adjusted for year of birth, gender, smoking status, and daily caffeine intake. None of the interactions were statistically significant.

All SNPs in controls conformed to Hardy-Weinberg equilibrium (p>0.05). None of the SNPs examined was related to PD risk under the additive models or dominant models; however, rs11136000 at the *CLU* locus was associated with PD risk under the recessive model (Table 2). After adjusting for year of birth, gender, smoking and caffeine intake, TT carriers had approximately 29% lower odds of having PD compared to participants with other genotypes (OR = 0.71, 95%CI: 0.55-0.92). Further adjustment for family history and *ApoE* ε4 status did not change the result (OR = 0.74, 95%CI: 0.57-0.95). Subgroup analyses generally showed similar results (Figure 1). The results were also similar for cases diagnosed before 2000 (OR = 0.76, 95%CI: 0.55-1.07) and for cases diagnosed after 2000 (OR = 0.69, 95%CI: 0.50-0.95). When PD cases with and without dementia were analyzed separately, the association appeared to be stronger for PD with dementia (recessive model OR = 0.49, 95%CI: 0.27-0.91) than for PD without dementia (OR = 0.81, 95%CI: 0.61-1.06); however, the associations were in the same direction and 95% CIs overlapped. In contrast to *CLU*, SNPs at the *PICALM* and *CR1* loci were not associated with PD risk in all analyses.

**Table 2:** Parkinson's disease in relation to SNPs associated with AD.

Models		CLU - rs11136000 *b/B = T/C		PICALM - rs3851179 *b/B = A/G		CR1 - rs6656401 *b/B = A/G	
Case/Control		785/1576#		775/1562#		782/1569#	
		OR (95%) CI	p	OR (95%) CI	p	OR (95%) CI	p
Additive	Model01	0.92(0.8101.04)	0.17	1.07(0.9401.21)	0.32	0.99(0.8401.16)	0.86
	Model 2	0.91(0.80 1.04)	0.18	1.08(0.95 1.23)	0.25	0.99(0.84 1.16)	0.88
Dominant/ (Bb+bb) vs BB	Model 1	1.00(0.84 1.20)	0.96	1.07(0.89 1.28)	0.48	0.96(0.80 1.16)	0.68
	Model 2	0.98(0.82 1.18)	0.86	1.10(0.91 1.32)	0.33	0.95(0.79 1.15)	0.60
Recessive/ bb vs (BB+Bb)	Model 1	0.71(0.55 0.92)	0.008	1.13(0.88 1.45)	0.34	1.15(0.71 1.87)	0.57
	Model 2	0.74(0.57 0.95)	0.02	1.13(0.88 1.45)	0.36	1.27(0.77 2.09)	0.35

*b: minor allele; B: major allele; ^#^ numbers of participants to be genotyped successfully.

Model 1: logistic models adjusted for year of birth, gender, smoking status, and daily caffeine intake.

Model 2: further adjusted for family history of PD and ApoE ε4 status (ε4 carrier and Non-ε4 carrier).

## Discussion

In this large case-control analysis, we found preliminary evidence that rs11136000 at the *CLU* locus might be associated with PD, and this association appeared to be stronger for PD patients with dementia. Our analyses also suggest that this relationship was independent of *ApoE* or known risk factors for PD.

Several clinical and pathological similarities between PD and AD have led to the hypothesis that these two conditions may share some pathogenic mechanisms or risk factors [1], [12]. *ApoE* ε4, the most established genetic risk factor for AD, has been examined in relation to PD in over 30 case-control studies. Most prior studies reported a null association between *ApoE* ε4 and PD [13], [14], although a few reported either higher or lower PD prevalence among ε4 carriers [15], [16], [17], [18]. We also examined *ApoE* ε4 in our study sample. We found that while *ApoE* ε4-carriers had a higher prevalence of dementia than non-carriers among PD patients, *ApoE* ε4 was associated with a statistically significant lower risk of PD (OR: 0.75, 95%CI: 0.59-0.94) [19]. Although this result needs confirmation from other studies, it is consistent with several previous epidemiological observations that higher plasma cholesterol was associated with a lower risk for PD [20], [21], [22].

Two recent large GWAS [9], [10] have independently indentified rs11136000 at the *CLU* locus as a risk allele for late onset AD, and the finding was subsequently confirmed in a large case-control study and a meta-analysis [11]. Clusterin may have diverse biological functions; however, the exact mechanisms how clusterin affects AD pathogenesis are yet to be unveiled [23]. Several hypotheses have been proposed, including actions on amyloid-β deposition or clearance, cell survival, neuroinflammation, or lipid trafficking [23], [24]. Some of these biological processes have also been implicated in PD pathogenesis. Direct explorations of potential roles of clusterin in Parkinson pathogenesis are rare. Preliminary evidence suggests that clusterin may affect the formation of Lewy body by acting as a chaperone [25], [26]. Further, a recent clinical study found that PD patients had significantly higher levels of clusterin in the cerebrospinal fluid than in controls [27].

An alternative explanation is that the finding was mediated by an association of *CLU* with PD dementia. In support of this, we found that the association was stronger for PD dementia than for PD without dementia, although both associations were in the same direction. The presence of dementia in our study was collected only as part of PD diagnostic confirmation and thus we were unable to examine this possibility in details. Therefore, if our finding is confirmed, future studies should also investigate the possibility that the association of *CLU* with PD might be explained by its potential association with dementia.

*CR1* [10] and *PICALM* [9] were each implicated in one of the above referenced AD GWAS. *CR1* encodes complement receptor 1, and may play a role in neuroinflammation and amyloid clearance [24]. On the other hand, *PICALM* encodes a ubiquitously expressed phosphatidylinositol-binding clathrin assembly protein which may affect synaptic neurotransmission and trafficking [24]. In our study sample, SNPs selected for these two genetic loci were not associated with PD risk. The later meta-analysis of AD GWAS data [11] found that rs3821179 at the *PICALM* locus was only associated with AD risk among *ApoE* ε4 carriers; our analysis did not reveal a significant interaction between rs3821179, or any other SNPs, and ApoE ε4 allele [6]. Unlike clusterin, for which there is preliminary experimental evidence, we are not aware of any evidence suggesting a link between *CR1* or *PICALM* and PD.

To the best of our knowledge, this is the first attempt to examine recent AD GWAS findings in PD pathogenesis. Therefore, our analyses should be considered exploratory and hypothesis generating; accordingly, we explored the associations under different genetic models and did not adjust for multiple comparisons. Although this study is among the larger population based genetic studies on PD, the statistical power was limited. For example, we had only an adequate statistical power for a common SNP (minor allele frequency ≥38.5%) and a moderate association (OR ≤0.69 or ≥1.38) under a recessive model. For this consideration, we only chose the most significant SNPs in previous AD GWAS. Further, it is important to point out that the previous AD GWAS analyses were based on additive model, but our analysis on rs11136000 was only statistically significant under recessive model. Due to these uncertainties, our finding on rs11136000 only suggests a potential link to this genomic locus; future studies with larger sample size and denser SNP coverage are needed to confirm our result, and if confirmed, further investigations would be needed to identify the underlying gene and functional SNPs.

In this study, we confirmed the diagnosis of PD with patients' treating physicians, rather than having the diagnosis uniformly made by study related movement disorder specialists. We therefore could not exclude the possibility of a few misdiagnoses. Nevertheless, recent clinicopathological studies showed a 90% accuracy for PD diagnosis made by clinical neurologists [28]. We were also concerned about the possibility that cohort participants with dementia might have been less likely to participate in this study than participants without dementia. This factor, however, might have similarly affected both cases and controls. Further, results from sensitivity analyses stratified by the year of PD diagnosis or baseline age showed similar results across subgroups. We therefore expect the impact from this bias is likely to be limited. With the same reasoning, we expect that poor health or poor survival of prevalent PD patients might also have limited impacts on the analysis. Finally, the current analysis was conducted among non-Hispanic Whites, therefore the generalizability of these findings to other ethnicities is uncertain.

## Methods

### Patients

PD cases and controls were from the Parkinson's, Genes, and Environment (PAGE) study that was nested within the large prospective cohort: the National Institute of Health (NIH)-AARP Diet and Health Study cohort (Formerly known as American Association of Retired Persons. [29]. This cohort was established in 1995-1996 for research on cancer and other chronic diseases and collected detailed dietary and lifestyle data at baseline [29]. Potential PD patients in this cohort were identified from self-reports in the cohort's follow-up survey in 2004-2006, which asked participants whether they had ever been informed by a doctor that they had PD. We then contacted surviving self-reported cases to verify the diagnosis and to collect saliva samples for genetic analysis. Detailed procedures for case confirmation were published previously [30]. Briefly, we first obtained confirmation and permission from self-reported PD patients and then contacted their treating physicians to complete diagnostic form and a copy of the relevant medical record. As part of this clinical data collection, we also collected information on the dates of first symptoms and diagnosis, the presence of dementia, and the family history of PD among first degree biological relatives. The Parkinson case was confirmed if the diagnosis was either validated by the patient's treating physician or by medical record review showing a final PD diagnosis or at least two cardinal signs of PD with one being resting tremor or bradykinesia, a progressive course, and the absence of unresponsiveness to levodopa or other features suggesting an alternative diagnosis. Only physician confirmed PD cases (86.7% by neurologist and movement disorder specialists) were included in the analysis. A similar procedure was successfully used in other large cohorts [31], [32], [33]. Potential controls were randomly selected from cohort participants who did not report PD in the follow-up survey, frequency matched to self-reported cases on year of birth, gender, and ethnicity. Saliva samples were obtained from cases and controls using Oragene™ saliva collection kits (DNA Genotek Inc., Ontario Canada). For controls, family history of PD was asked as part of the saliva collection. A total of 838 physician confirmed PD cases and 1,703 controls were genotyped for the current analyses. To avoid population stratification, we excluded 30 cases and 80 controls who were not non-Hispanic Whites or had missing information on ethnicity. We further excluded 17 cases and 43 controls who failed genotyping on all three SNPs, leaving us a total of 791 cases and 1, 580 controls for the final analyses. The study protocol was approved by the Institutional Review Board of the National Institute of Environmental Health Sciences with written consent from all study participants.

### Exposure Assessment

The baseline questionnaire of the NIH-AARP Diet and Health Study collected detailed data on diet, lifestyle, and population demographics, including birth date, gender, and ethnicity (Non-Hispanic White, Non-Hispanic Black, Hispanic, Asian, Pacific Islander, or American Indian/Alaskan Native) [29]. In addition, we asked participants to report whether they had ever smoked more than 100 cigarettes during their lifetime; and for “ever smokers”, we further asked for the typical amount of smoking, current smoking status and years since last smoking. Daily caffeine intake was derived from the baseline dietary survey.

### DNA extraction and genotyping

DNA was extracted from saliva samples collected with the Oragene™ collection kits. Three SNPs were selected based on their statistical significance as reported in the GWAS, one for each gene region: rs11136000 for CLU, rs6656401 for CR1, and rs3851179 for PICALM. One additional SNP on PICALM was also selected based on information from ALZgene.com; this SNP is in linkage disequilibrium with rs3851179 and the results were also similar, so we did not present detailed data for that particular SNP. Genotyping was performed by BioServe Biotechnologies, Ltd., Beltsville, MD, using MassARRAY iPLEXTM platform (Sequenom, San Diego CA). Sequenom is a PCR and mass spectrometry based system. Genotyping assays are custom developed using Mass ARRAY Assay Design 3.1 software. For quality control purpose, we randomly included 51 duplicated saliva DNA samples which were kept blinded to the genotyping laboratory; the genotyping reproducibility was 100%.

### Statistical analyses

We examined Hardy-Weinberg equilibrium with chi-square statistics. Odds ratios (ORs) and 95% confidence intervals (CIs) were derived from logistic regression models, adjusting for year of birth (in four groups), gender, smoking status (never, former, current) and daily caffeine intake (quintile). We also further adjusted for family history of PD and ApoE ε4 status. We examined each individual allele in relation to PD first under an additive model and then under dominant and recessive models. We began the analyses with all participants, and then stratified by individual covariates: year of birth (below or above median), gender (men vs. women), smoking status (never vs. ever smokers) caffeine intake (below or above median), family history of PD (Yes vs. No), and ApoE ε4 status (ε4 carriers vs. non- ε4 carriers).

To examine the potential influence of bias stemming from the possibility that PD cases with dementia were less likely to participate in the study than PD cases without dementia, we conducted a sensitivity analysis to examine the association of genes with PD separately for cases diagnosed before the year 2000 and diagnosed after the year 2000. We assumed that cases with longer duration of PD (e.g. diagnosed before 2000) were more likely to have dementia than cases with shorter duration, and therefore such a bias would affect the analyses for cases with years of PD more so than for recent cases. With the same reasoning, stratified analysis by age may also help clarify the potential influence of dementia as the prevalence of dementia increases dramatically with age. Finally, we also analyzed rs11136000 at the CLU locus separately in relation to PD with dementia and PD without dementia. Statistical analyses were performed using SAS version 9.2 (SAS Institute Inc, Cary, NC) combined with Plink v1.07. Post-hoc power calculation was performed with Quanto V1.2.4 [34]. Two sided p<0.05 was considered statistically significant.
